# Communication breakdown: Limits of spectro-temporal resolution for the perception of bat communication calls

**DOI:** 10.1038/s41598-021-92842-4

**Published:** 2021-07-01

**Authors:** Stephen Gareth Hörpel, A. Leonie Baier, Herbert Peremans, Jonas Reijniers, Lutz Wiegrebe, Uwe Firzlaff

**Affiliations:** 1 Chair of Zoology, School of Life Sciences, Technical University of Munich, Liesel-Beckmann-Str. 4, 85354 Freising, Germany; 2 Neurogenetics of Vocal Communication Group, Max Planck Institute for Psycholinguistics, Nijmegen, The Netherlands; 3 Department Biology II, Ludwig Maximilians University Munich, Großhaderner Strasse 2, 82152 Martinsried, Germany; 4 Department of Engineering Management, Faculty of Business and Economics, University of Antwerp, 2000 Antwerp, Belgium

**Keywords:** Cognitive neuroscience, Auditory system, Cortex

## Abstract

During vocal communication, the spectro-temporal structure of vocalizations conveys important contextual information. Bats excel in the use of sounds for echolocation by meticulous encoding of signals in the temporal domain. We therefore hypothesized that for social communication as well, bats would excel at detecting minute distortions in the spectro-temporal structure of calls. To test this hypothesis, we systematically introduced spectro-temporal distortion to communication calls of *Phyllostomus discolor* bats. We broke down each call into windows of the same length and randomized the phase spectrum inside each window. The overall degree of spectro-temporal distortion in communication calls increased with window length. Modelling the bat auditory periphery revealed that cochlear mechanisms allow discrimination of fast spectro-temporal envelopes. We evaluated model predictions with experimental psychophysical and neurophysiological data. We first assessed bats’ performance in discriminating original versions of calls from increasingly distorted versions of the same calls. We further examined cortical responses to determine additional specializations for call discrimination at the cortical level. Psychophysical and cortical responses concurred with model predictions, revealing discrimination thresholds in the range of 8–15 ms randomization-window length. Our data suggest that specialized cortical areas are not necessary to impart psychophysical resilience to temporal distortion in communication calls.

## Introduction

Vocal communication is a key feature in the behaviour of many vertebrate species, especially in birds and mammals^1^. Although the degree of sophistication varies across taxa, a common challenge in vocal communication is to detect and interpret relevant signals. Studies that investigate the sensory processing of vocal communication signals, including human speech, typically make use of systematic degradations of the vocalizations’ spectral and/or temporal structure. Such degradation is achieved either by reversing the temporal structure of the signal under test^2–4^ or by a more complex manipulation of the signal’s spectro-temporal content^5^.

In a study on human speech perception, Saberi and Perrott^6^ (see also Steffen and Werani^7^) demonstrated perfect intelligibility of a degraded spoken sentence when time-reversal-segment lengths were < 50 ms. Thus, speech signals had to be analysed with integration time on this scale to ensure efficient decoding. These findings were considered to support the notion that a precise analysis of the local acoustic spectrum is non-essential to the speech code^6,8,9^. On the contrary, global cues such as slow modulation envelopes in the order of 3–8 Hz might be crucial to speech intelligibility in humans and may be the reason for the resilience of speech to distortion of short segments (< 50 ms)^6,9,10^.

Bats are known to rely on meticulous temporal coding. They use an elaborate biosonar system^11,12^ where the precise representation of the time delay between outgoing ultrasonic pulses and returning echoes is crucial to catch prey in agile flight^13^. In addition to their active acoustic orientation, bats also display a large repertoire of complex communication sounds^14–17^ and are considered vocal learners^18–21^. In bats’ auditory cortical areas where echo-delay information is processed, single neurons also show robust responses to species-specific communication calls^16^. Further, these responses are sensitive to manipulation of calls in the time domain. We therefore hypothesized that specialized tuning for temporal precision shaped by the requirements of bat biosonar might serve as a pre-adaptation for high resilience to spectro-temporal distortion in social vocalizations, as even short, intact segments should convey enough vital information for call recognition. Consequently, we predicted low thresholds for the detection of spectro-temporal distortions in communication calls of echolocating bats. This may hold true especially in agonistic contexts, where communication often plays a critical role. When two animals compete for a resource, failure to detect or correctly interpret an aggression call may immediately escalate a conflict and lead to costly physical fighting^1^.

Here we investigated the limit of spectro-temporal distortion that can be tolerated for call discrimination in the bat *Phyllostomus discolor*. We systematically distorted the spectro-temporal structure of the calls by means of phase-randomization and determined the degree of spectro-temporal distortion that facilitated the recognition of the original, undistorted calls. To explore the origin of call recognition limits, we modelled the bat auditory periphery and tested model predictions with experimental data. In concurrence with model predictions, both behavioural and cortical responses revealed high spectro-temporal resolution, with thresholds in the range of 8–15 ms randomization-window length.

## Results

### Spectro-temporal distortion of bat calls

**Figure 1:**
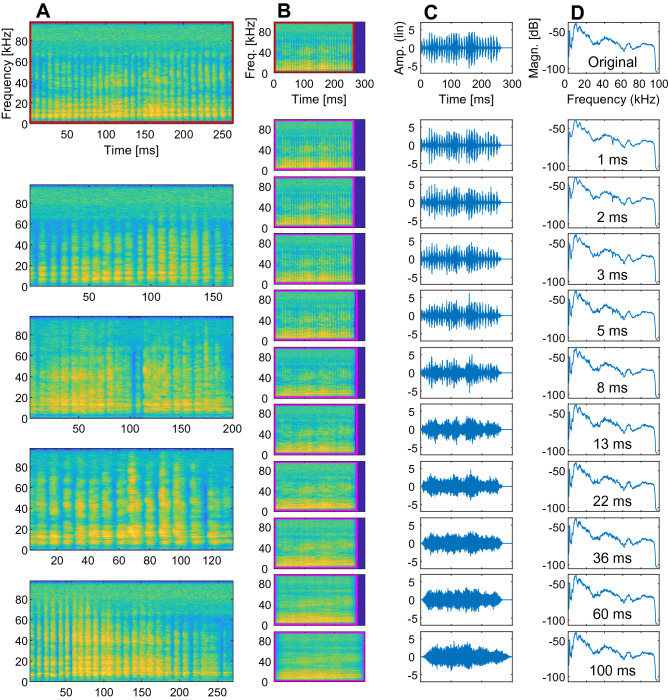
Original and phase-randomized aggression calls. (**A**) The spectrograms of the five original aggression calls used in this study illustrate strong amplitude modulations (FFT size 1024 samples, overlap 1020 samples). **(B**) The spectrograms of one of the original calls (highlighted in red) and its ten phase-randomized versions (highlighted in magenta) exemplify the effect of increasing randomization-window lengths ranging from 1 to 100 ms (top to bottom, cf panel (**D**). (**C**) The waveforms of the signals in (**B**) illustrate the decrease in envelope amplitude fluctuations with increasing randomization-window length (top to bottom). All calls were normalised to RMS amplitude. (**D**) The magnitude spectra of the signals in (**B**) reveal no strong effect of the phase-randomization. Numbers depict randomization-window lengths.

**Figure 2:**
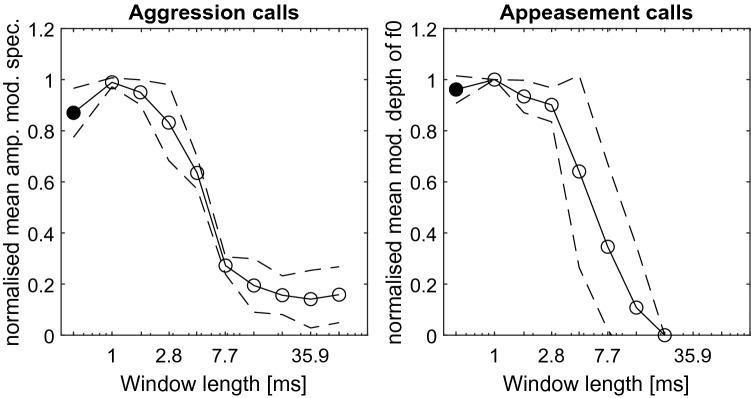
Phase-randomization changes temporal and spectral modulation cues. Left: The spectrum of amplitude-modulation rates in the aggression calls changes as a function of phase-randomization window-length. Peak magnitudes for original calls (filled circle) and respective distorted versions (circles) were normalised and averaged across the five aggression calls. Right: The depth of SFM of the fundamental frequency f_0_ in the appeasement calls changes as a function of phase-randomization window-length. Peak SFM depth for f_0_ of original calls (filled circle) and respective distorted versions (circles) were normalised and averaged across the five appeasement calls. Dashed lines depict the standard deviation. Note that AM rate spectrum and SFM depth are exemplary technical measures to visualize the distortion of modulation patterns and should therefore neither be directly compared with each other nor to the psychometric functions.

The spectro-temporal information in two groups of communication calls of *Phyllostomus discolor*, aggression calls (Fig. 1A) and appeasement calls (Figure S1A), was systemically distorted by means of phase-randomization in windows of increasing length (Figs. 1B–D, S1B–D). This manipulation effectively disrupts the amplitude modulation (AM) pattern characteristic to aggression calls (Fig. 1B, top to bottom) and the sinusoidal frequency modulation (SFM) pattern characteristic to appeasement calls (Figure S1B, top to bottom), respectively. Quantification of the amount of distortion of the modulation patterns reveals the following: In the aggression calls, the spectrum of amplitude modulation rates changes as a function of phase-randomization window-length (Fig. 2 left). For window-lengths > 2 ms, the peak magnitude in the spectrum of the distorted calls (circles) drops far below the peak magnitude for original calls, thus phase-randomization using these longer window-lengths effectively disrupts amplitude modulation in these calls, i.e., their temporal envelopes. In the appeasement calls, the depth of SFM of the fundamental frequency f_0_ (and thereby all harmonic frequencies) also changes as a function of phase-randomization window-length (Fig. 2 right). The SFM depth of the distorted calls rapidly drops with increasing window-length (> 2 ms). Hence, phase-randomization also effectively disrupts spectral modulation patterns in appeasement calls.

### Modelling of the bat auditory periphery

**Figure 3:**
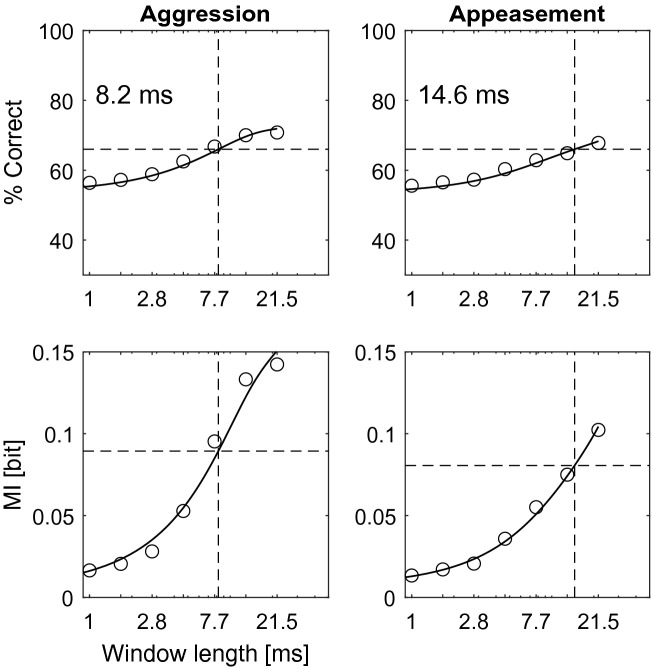
Simulated discrimination performance of the auditory periphery. Top row: Simulated psychometric functions reveal that aggression calls (left) can be discriminated at a shorter phase-randomization window-length than appeasement calls (right). Vertical dashed lines mark the discrimination thresholds of 8.2 and 14.6 ms, respectively. Horizontal dashed lines at 66% correct depict significance level (binomial test, 50 trials, *p* < 0.01). Bottom row: With increasing phase-randomization window-length, the change in Mutual Information (MI) content is greater for aggression calls (left) than for appeasement calls (right). Horizontal dashed lines mark the MI content of the calls at respective threshold window-lengths (vertical dashed lines).

We evaluated the auditory periphery’s performance in discriminating original versions of calls from increasingly distorted versions of the same calls. We modelled the performance of the bats’ peripheral auditory processing as a function of phase-randomization window-length (Fig. 3). This model generated a call’s spectro-temporal representation as a function of tonotopic frequency and time (Figure S2) and compared these so-called auditory spectrograms of original calls with auditory spectrograms of the respective phase-randomized versions. The model’s discrimination performance yielded a threshold window-length of 8.2 ms and 14.6 ms for aggression and appeasement calls, respectively (Fig. 3).

Are the observed spectro-temporal resolution limits related to information that is contained in the different calls? To answer this question, we calculated the information content as a function of phase-randomization window-length, with “information” not referring to any semantical information that may be inherent to the calls, but strictly to Shannon’s Mutual Information $MI$^22^. $MI$ is calculated using the probability $P_{R}$ that the model makes a correct discrimination decision based on the two calls it receives as input(see “Methods” section) and quantifies the amount of information the calls contain about the discrimination decision. This information is equal to the reduction in uncertainty of the discrimination decision due to knowledge of both the original and the distorted call. Therefore, as longer phase-randomization window-lengths influence the spectro-temporal properties of the calls more, we expect $MI$ to increase with increasing window length. Indeed, with increasing distortion of a call relative to the original call discrimination between the original and the distorted call becomes easier, making the discrimination decision more predictable, i.e., less uncertain. Using the model’s discrimination threshold (Fig. 3), we can derive that significant discrimination is possible when $MI$ exceeds ~ 0.1 bits (in the psychophysical paradigm, the maximum amount of *MI* available is 1 bit as bats have to decide between two equally probable options, left or right; see next section).

### Psychophysics

We tested the model predictions with two *P. discolor* bats in a psychophysical 2-alternative, forced-choice (2AFC) paradigm. We tested the bats with both aggression and appeasement calls (Figs. 1 and S1). Aggression calls were longer than appeasement calls (134 to 270 ms vs. 51 to 56 ms), had a lower fundamental frequency (~ 7.5 kHz vs. ~ 17 kHz) and pronounced amplitude-modulations (modulation rate ~ 100–150 Hz) instead of sinusoidal frequency-modulations (modulation rate ~ 70–90 Hz).

Both bats learned to discriminate between an unaltered call and a phase-randomized version of that same call for both aggression and appeasement call stimuli. We used the behavioural response of the bats to assess the spectro-temporal resolution limit, recording one psychometric function per bat. Each psychometric function yielded a threshold window-length, i.e., the randomization-window length that still let the bat identify the natural signal version, as a proxy for auditory spectro-temporal resolution limit. The thresholds were extracted from a cubic spline fit to the psychometric functions (see “Methods” section).

**Figure 4:**
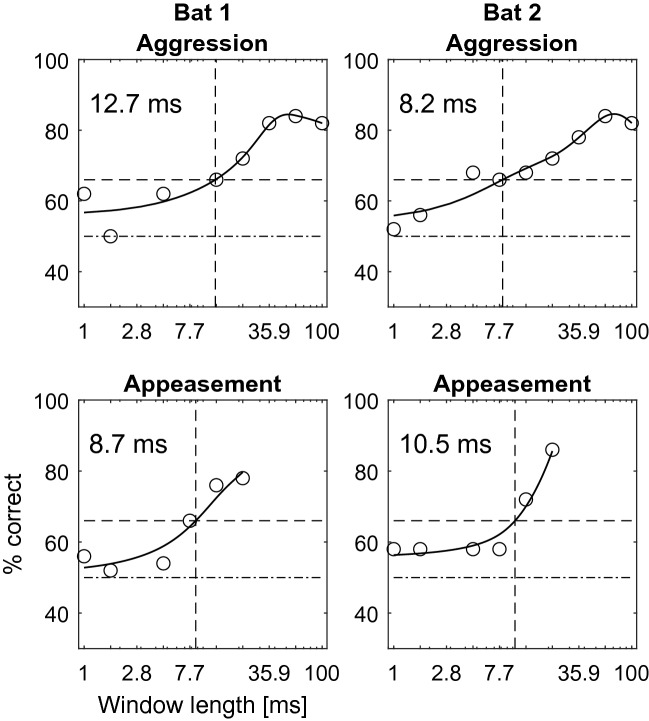
Psychometric functions of discrimination performance. For aggression calls, the psychometric functions yield threshold window-lengths of 12.7 and 8.2 ms for Bat 1 and Bat 2, respectively. For appeasement calls, the psychometric functions yield threshold window-lengths (vertical dashed lines) of 8.7 and 10.5 ms for Bat 1 and Bat 2, respectively. Note that the maximum window length for appeasement calls is 21.54 ms, owing to the short duration of these calls. Each circle marks a bat’s discrimination performance across 50 trials. Horizontal dashed lines at 50% and 66% correct depict chance and significance level (binomial test, 50 trials, *p* < 0.01), respectively.

Both bats reliably (> 70% correct choices; Fig. 4) identified the original call when phase-randomization windows were very long (21.6 ms and longer). In contrast, when windows were very short (1.7 ms and shorter), both bats failed the discrimination task and their performance dropped to chance level. The discrimination performance between bats did not differ significantly in a paired t-test (aggression calls: t(7) = 0, *p* = 1; appeasement calls: t(5) = 0.53, *p* = 0.62). The obtained threshold window-lengths of bat 1 and bat 2, respectively, were 8.2 ms and 12.7 ms for aggression calls and 10.5 ms and 8.7 ms for appeasement calls (66% correct; binomial test, 50 trials, *p* < 0.01; Fig. 4).

### Neurophysiology

**Figure 5:**
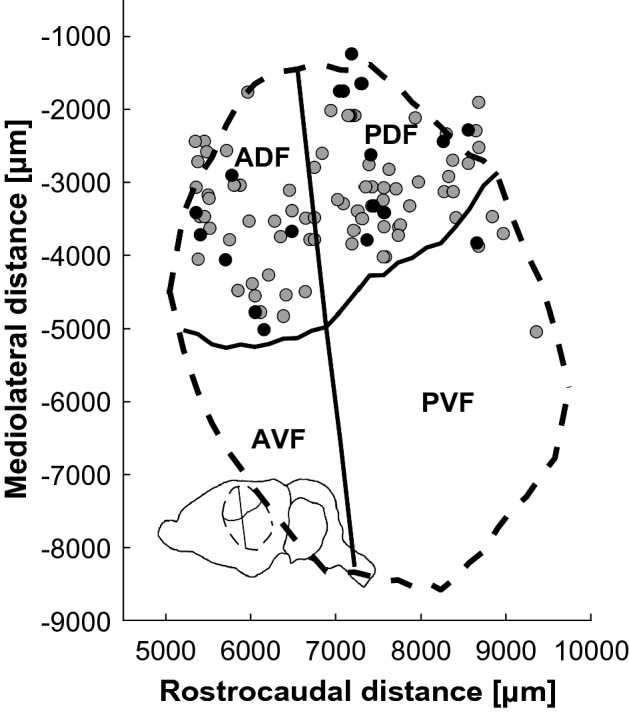
Recording sites and subfields in the auditory cortex (AC) of *P. discolour*. Projections of recording sites (gray & black circles) on an unrolled and flattened cortical surface with schematic AC subfields: anterior dorsal field (ADF), posterior dorsal field (PDF), anterior ventral field (AVF) and posterior ventral field (PVF). Units that showed a significant response rate decrease are marked by black circles. Inlay shows overall position of the AC in the brain of *P. discolor*.

Recordings stem from the data-set published by Hörpel and Firzlaff^23^, but none of the analyses and results presented here are part of this former publication. There, we recorded the responses of auditory cortical (AC) neurons in anaesthetised *P. discolor* to both unaltered aggression calls and their respective phase-randomized versions. In total, we recorded from 114 cortical response units (consisting of 1–3 neurons, hereafter “units”) in both hemispheres of three females and one male bat. The majority of the units were located in layers III–V of the anterior (ADF) and posterior dorsal field (PDF, nomenclature following^24^) (Fig. 5). All units responded well to pure tones in the frequency range (5–60 kHz) of the *P. discolor* calls used in this study (cf. Fig. 1D).

**Figure 6:**
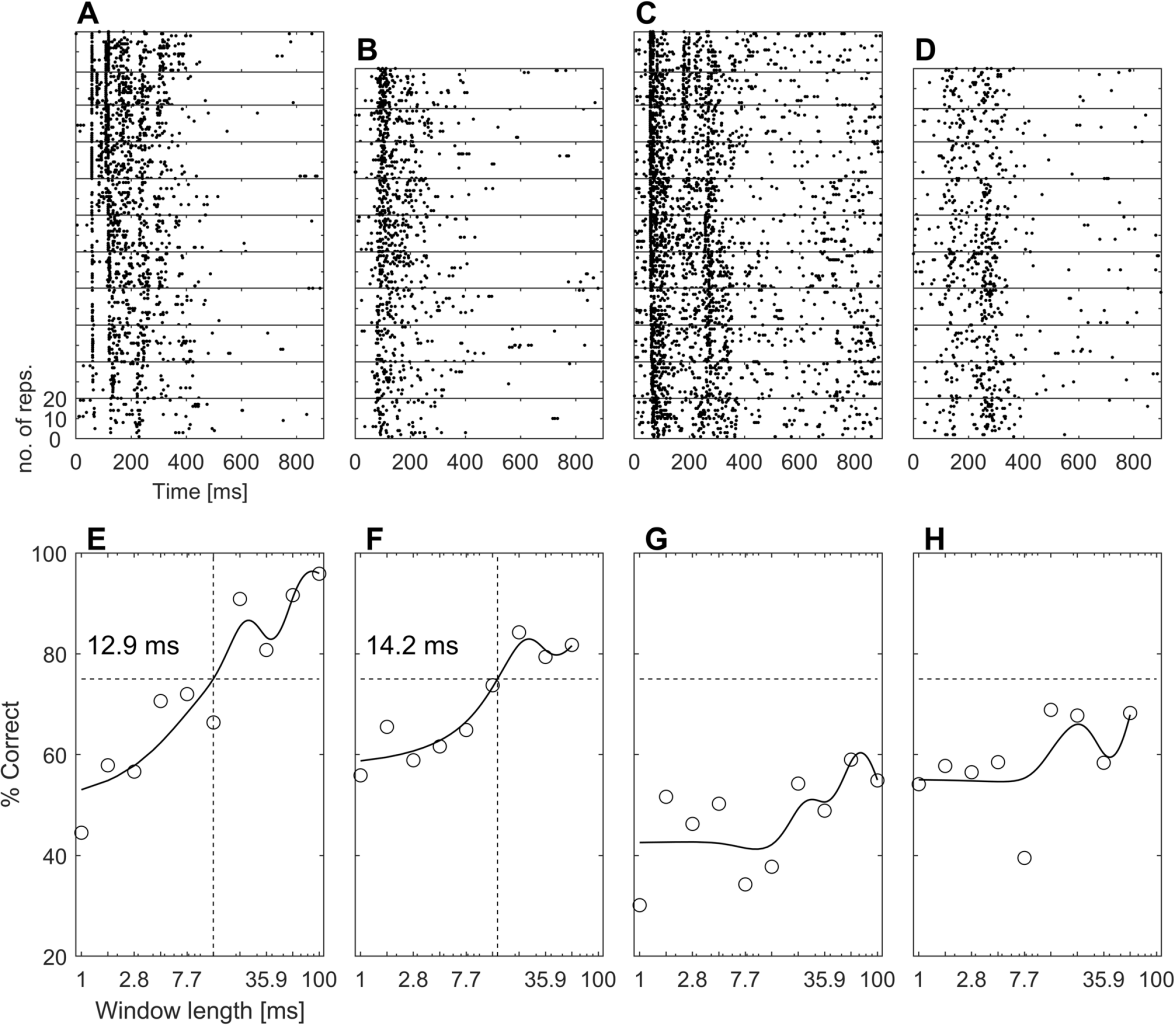
Raster plots and neurometric functions of four exemplary cortical neurons presented with phase-randomized aggression calls. (**A–D)** Each block within the spike raster plot shows neuronal responses to one (increasingly phase-randomized) version of the stimulus. For the topmost block, the original call was presented, and for the bottommost block, the respective degraded version with the longest phase-randomization window was presented, respectively. Note that the number of phase-randomization steps varied with call length. Each stimulus version was presented 20 times in total. The unit in A and C shows a distinct temporal response pattern, the unit in (**B,D**) shows a more tonic response pattern. (**E,F)** Neurometric functions as obtained from ROC analysis show significant discrimination performance by the two units from A-B and yield discrimination thresholds of 12.9 ms and 14.2 ms, respectively. (**G,H)** Neurometric functions show no significant discrimination performance by the two units from (**C**,**D**).

We analysed response properties of the units to acoustic stimulation with aggression calls (Fig. 6, top row). In 18.4% of units (**21/114**), response rates significantly decreased with increasing phase-randomization window length (Fig. 6A,B; *p* < 0.05, Kruskal–Wallis-nonparametric one-way ANOVA), whereas in 74.6% of units (**85/114)**, phase–randomization of calls did not evoke significant changes in response rate (Fig. 6C,D).

Only few units (**8/114, 7%**) showed an increased response rate with increasing phase-randomization window length (*not shown*). Responses of cortical units showed either a distinct temporal pattern or a more tonic response to non- phase-randomized calls which often degraded for phase-randomized versions (Fig. 6A–D). The changes in the firing rate were observed in both groups of units with either of these temporal response patterns.

**Figure 7:**
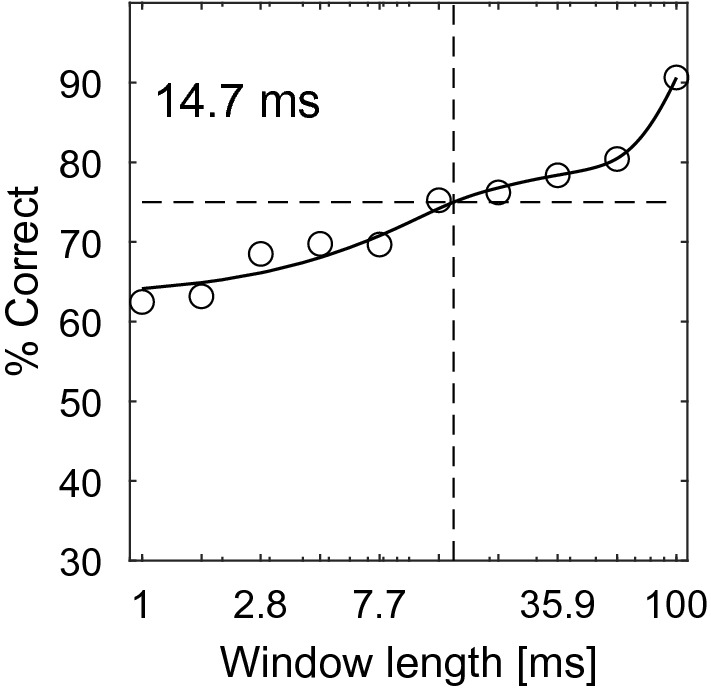
Cortical discrimination performance in response to phase-randomized aggression calls. Each circle marks the population discrimination performance of the 21 cortical units that showed a significant decrease in their firing rate when presented with increasingly phase-randomized aggression calls. The resulting function yields a threshold window-length of 14.7 ms (vertical dashed line). The horizontal dashed line at 75% depicts significance level (20 trials, binomial test, *p* < 0.01).

To compare the neural responses with both the model and psychophysical responses, we generated neurometric functions (Fig. 6, bottom row). This was done by means of a receiver operating characteristics (ROC) analysis, comparing the neuronal response to the original stimulus with the neuronal responses to each increasingly phase-randomized version (see “Methods” section). For units in which response rates did not significantly change, neurometric functions did not reach the significance threshold (Fig. 6G,H). For most units with significantly decreased response rates (**15/21, 71.4%**), their neurometric functions crossed the significance threshold of 75% correct. That is, these units’ response allowed for discrimination between a phase-randomized version of a call and the original one (Fig. 6E,F). For few individual units (**4/15, 26.7%**), correct discrimination was possible at a very short phase-randomization window-length of 1 ms. However, for most units (**11/15, 73.3%**) correct discrimination was only possible at a long window length of 59.9 ms. Overall, the discrimination-performance threshold of this subpopulation of cortical units was 14.7 ms (Fig. 7). Units showing either significant or non-significant neurometric functions were randomly distributed within cortical fields (Fig. 5).

## Discussion

Spectro-temporal integrity of vocalizations is crucial for vocal communication. We have determined the degree of distortion that impedes the recognition of communication calls in the bat *Phyllostomus discolor*. With a model of the auditory periphery and concurrent experimental data, we show that spectro-temporal resolution is high, demonstrating that bats possess a high resilience to distortion in social vocalizations.

In *P. discolor,* the auditory periphery is not highly specialized for echolocation unlike in other bats^25^ and therefore well comparable to those of other small mammals. Previous work on both mammals and birds supports our findings that a model of the non-specialized auditory periphery suffices to predict higher-level discrimination thresholds: a simple spectrogram-based cochlear model explained neuronal responses in the ferret primary auditory cortex as well as more complex, biologically detailed cochlear models did^26^. A study using spectro-temporally degraded bird song as stimuli found spectro-temporal resolution in HVC neurons of up to 2 ms or 500 Hz^5^. This resolution matches the spectro-temporal resolution of auditory filters in the bird auditory periphery^27^, suggesting that bird song is well represented by amplitude envelopes extracted in the periphery. The above line of argument makes a general pre-adaptation of the auditory system unlikely, i.e. a pre-adaptation of the auditory system as a whole towards the processing of fast spectro-temporal modulations in communication calls would be generally beneficial for vocal communication and vocal learning in bats. This would be in contrast to the growing body of literature on bats in the context of vocal learning^19,20,28,29^, where bats are indeed discussed as being pre-adapted for vocal learning as a consequence of their well-developed biosonar system, which requires fast temporal auditory processing and sensory-motor feedback loops for call-production control. However, our findings do not exclude the possibility of an existing pre-adaption in the context of vocal-learning abilities of bats, per se. Even though the performance of the auditory periphery would be sufficient to predict behavioural performance, it should be feasible to re-map call features such as spectro-temporal modulation on the level of the auditory cortex. This would be in line with concepts of neural processing along the ascending auditory pathway^30^. Furthermore, neuronal sensitivity to spectro-temporal changes must be high in neurons involved in the recognition of complex vocalizations, as previously discussed^5^. It is therefore intriguing that the cortical units sensitive to phase-randomization window-length in our study were located in the dorsal fields of the auditory cortex. These fields contain neurons which are sharply tuned to echo-delay with high temporal resolution, both in our experimental animal^31^ and other bat species^32^.

Our current findings are supported by several earlier studies^16,33–35^. For example, temporal manipulation of calls effectively changed responses of neurons in the FM-FM area in the cortex of the bat *Pteronotus parnellii*^16^. In addition, neurons in the dorsal auditory cortex can encode amplitude modulations up to unusually high modulation rates with high fidelity, corresponding to the short phase-randomization window lengths in this study^23,36^. However, given their known multifunctional roles, it would go too far to infer a sole specialization of such areas for either echolocation or call processing.

The question whether call-specific neurons exist that might selectively encode spectro-temporal distortions of certain calls or call types cannot be answered by our study.

However, one could argue that a strong specificity to single calls would not be required. While call-specific neurons in the auditory cortex of mammals have been reported^37,38^, coding of slow modulation envelopes might be more important than call selectivity to represent animal vocalizations on a neuronal level^3,4^. In songbirds, song-specific neurons have been reported mainly in the neural network involved in vocal imitation and production (reviewed e.g. in^39,40^). Only few studies report such a selectivity in non-primary and primary regions of the bird auditory forebrain after training to learn new vocal elements (e.g.^41,42^). Should mammalian cortical regions involved in vocal production learning be identified in the future, we could expect to observe more specialized neuronal responses to the stimuli used here. Good candidate regions would be located in the frontal cortex, as a part of the cortico-striatal pathway of vocal production learning (e.g.^43^).

Our model simulates the auditory periphery of *P. discolor* up to the level of the auditory nerve^44–46^. It performs an envelope extraction in the different frequency bands. Phase-randomization distorts the envelope, leading to a drop in model performance. The aggression calls used in this study are typically characterised by strongly amplitude-modulated (AM) elements with modulation rates in the range of 100–150 Hz^23^. For this call type, the model predicts a discrimination threshold of 8.2 ms phase-randomization window length, which corresponds to a modulation rate of 122 Hz. In contrast to the aggression calls, the appeasement calls used here show merely shallow amplitude modulations; instead, they are characterised by strong sinusoidal frequency modulations (SFMs) with modulation rates of ~ 70–90 Hz (Fig. S1A). For appeasement calls, the model predicts a threshold of 14.6 ms phase-randomization window length, corresponding to a modulation rate of 68 Hz. As a matter of fact, phase-randomization with increasing window length as applied in our study disrupts the characteristic modulation patterns (Fig. 2).

The notion that bats’ discriminatory performance depends mainly on the modulation properties of the calls is supported by the results of the model’s information analysis. We assume that a bat needs a certain amount of information *MI* to make its decision. The rise in $MI$ as a function of phase-randomization window-length is steeper for aggression calls than for appeasement calls (Fig. 3, bottom row), indicating that phase randomization has a more pronounced effect on the cues used by the bats to recognise aggression calls (see Fig. 2) and supporting the notion of the discriminatory performance depending on modulation features. Indeed, higher values for the *MI* means that more information is available to the bat for discriminating between the original and the distorted call, i.e., less of the cues used by the bat to *recognize* the original call are still present.

We found that behavioural discrimination thresholds were within the same order of magnitude as the ones derived from the model. However, given our sample size for the psychophysical experiment, we can neither confirm nor refute the difference between thresholds in response to aggression and appeasement calls as it would be explained solely by modulation rate of the calls. In Bat 1, the threshold in response to appeasement calls was slightly lower than in response to aggression calls (8.7 vs. 12.7 ms, Fig. 4 left), whereas in Bat 2, it was slightly higher (10.5 vs. 8.2 ms, Fig. 4 right). We can speculate that with a higher sample size, the overall behavioural thresholds might eventually reflect the relationship between discrimination performance and call modulation properties more accurately.

Generally, frequency and amplitude modulations seem to be a prevalent feature of bat communication calls used in both agonistic and antagonistic social context as well as in distress context^15,47–49^. Auditory brainstem neurons in *P. discolor* respond to amplitude-modulated stimuli already at an early age^50^, further emphasizing the importance of these sounds for social interaction in bats.

Typically, animal vocalizations are selected for detectability in the animals’ respective acoustic environment^51^. Like all bats, *P. discolor* is a highly gregarious species that lives in large colonies of up to several hundred individuals^52^, therefore, overcoming noise is behaviourally highly relevant for them. Increasing the length of a vocalization is a known mechanism in birds and mammals including bats to prevail in noisy environments^53–55^. However, bats decrease complexity of their vocalization in response to noise^56^. Structuring communication calls by short, distinct acoustic features might be an overlooked strategy to ensure efficient communication.

In conclusion, our study highlights the perceptual importance of high-frequency envelopes in bat communication calls. A model based on the representation of bat communication calls in the auditory periphery predicts the cortical and behavioural responses. Our results demonstrate high spectro-temporal resolution in the range of 8–15 ms and suggest that the spectro-temporal integrity of characteristic acoustic features of communication calls, such as amplitude and/or frequency modulation, determines the discrimination performance. We speculate that temporal envelope features as they are extracted by the auditory periphery allow for high resilience to temporal distortion in social vocalizations.

Due to this, bats should gain an advantage for transmitting information in noisy environments and therefore bat communication should become more tolerant against masking by biotic or abiotic noise. We claim that in addition to slow-envelope coding, coding of fast temporal envelopes in segments of broken-down calls should be equally important in vocal communication for bats.

## Methods

### Experimental animals

The neotropical bat *Phyllostomus discolor* (family: Phyllostomidae) uses short (< 4 ms) downward modulated, multiharmonic calls in the frequency range between 45 and 100 kHz for echolocation^57^. In addition, *P. discolor* has a rich vocal repertoire for social communication, with up to twelve different classes of communication calls^15^. Here we used individuals originating from a breeding colony in the Department Biology II of the Ludwig Maximilians University (LMU) Munich. For the psychophysics part, we used two adult male individuals. For the neurophysiology part, we used one adult male and three adult female individuals. Husbandry details can be found in previous studies^23,58^. All experiments complied with the principles of laboratory animal care and were conducted under the regulations of the current version of the German Law on Animal Protection (approvals ROB-55.2-2532.Vet_02-13-147 and 55.2-1-54-2532-34-2015,), complying with the ARRIVE guidelines^59^. The study is approved by the ethics committee of the Regierung von Oberbayern (Committee following § 15.1 TierSchG, (German animal welfare law)).

### Stimuli

We selected two sets of communication calls from a library of 269 calls recorded in the *P. discolor* colony at the LMU Munich (cf.^23^). The first set (Fig. 1A) contained five calls that showed strong amplitude modulations (AM) but no frequency modulations and had a low fundamental frequency of approximately 7.5 kHz. These calls resembled the “HE” (high entropy) calls^15^ and show similarities to typical aggression and distress calls of other bat species^48,49^. They ranged in duration from 134 to 270 ms. We refer to this first set as “aggression calls”. The second set of stimuli (Figure S1A) contained five calls that showed shallow amplitude modulations and pronounced sinusoidal frequency modulations (SFM), a fundamental frequency of approximately 17 kHz, and a strongly harmonic spectrum. They ranged in duration from 51 to 56 ms. These calls have been classified as contact calls^15^ and we refer to this second set as “appeasement calls”.

For each of the five calls per stimulus set, we created either seven, nine or ten (depending on individual stimulus duration) increasingly phase-randomized versions. To do so, we first divided each call into windows with window lengths being the same inside a call and increasing across call versions. The length of windows ranged from 1 ms to either 21.6, 59.9 or 100 ms, depending on the overall call duration, with logarithmic spacing. We then randomized the phase spectrum inside each window (Matlab 2016b; MathWorks, Natick, USA). In contrast to human-speech studies, a simple time reversal of the signal inside a window was discarded as a viable method of signal disruption because of the highly periodic nature of the signals (a reversed amplitude modulation is still an amplitude modulation). Because we manipulated the phase spectrum, temporal information about frequency and envelope was increasingly altered with increasing randomization-window length (Figs. 1B,C and S1B,C), whereas the overall frequency content (i.e., the spectral envelope) was not affected (Figs. 1D and S1D). The original call and the phase-randomized versions were normalised based on their root mean square amplitude (RMS).

Our manipulation of the communication calls resulted in two stimulus sets consisting of a total of 53 and 40 stimuli, respectively: Set 1 consisted of two original aggression calls with nine phase-randomized versions each (window lengths 1.00, 1.67, 2.78, 4.64, 7.74, 12.92, 21.54, 35.94, and 59.95 ms), and three original aggression calls with ten phase-randomized versions each (window lengths 1.00, 1.67, 2.78, 4.64, 7.74, 12.92, 21.54, 35.94, 59.95, and 100 ms). Set 2 consisted of five original appeasement calls with seven phase-randomized versions each (window lengths 1.00, 1.67, 2.78, 4.64, 7.74, 12.92, and 21.54 ms). For the modelling and psychophysical experiment, both stimulus sets were used, for the neurophysiological experiment, only Set 1 (aggression calls) was used.

To quantify the distortion of modulation patterns present in both call types, we analysed the amplitude modulation (AM) strength and the sinusoidal frequency modulation (SFM) depth for aggression calls and appeasement calls, respectively. For aggression calls, the call envelopes were derived using Hilbert transformation and subsequent low-pass filtering at 500 Hz with a 2nd-order Butterworth filter. Then, AM spectra were determined with a fast Fourier transform (FFT) and peak magnitudes in the spectrum were normalised and averaged across the five calls. In appeasement calls, the SFM depth was analysed by tracking the fundamental frequency (f_0_) using the YIN algorithm^60^. Modulation depths were normalised and averaged across the five calls.

### Modelling

#### *Modelling discrimination performance *via* auditory spectrograms*

We simulated the discrimination of phase-randomized *P. discolor* communication calls using a physiologically plausible model of the bats’ peripheral auditory processing^44–46^. This simulation generated the so-called ‘auditory spectrogram’, a spectro-temporal representation of the calls as a function of tonotopic frequency and time^61^.

The simulation consisted of multiple stages (Figure S2): (1) The transfer characteristics of the bat middle ear were implemented with a broad band-pass filter (1st-order Butterworth, 10–50 kHz). The spectral range of the filter was designed to coincide with auditory thresholds found in *P. discolor* and other *Phyllostomid* bats^25,62^ and followed the frequency–response characteristics of a *Yangochiropteran* tympanic membrane (*Eptesicus pumilis*^63^). (2) The frequency-to-place conversion of the inner ear was emulated with a series of 4th-order gamma-tone filters (Figure S3)^64^. The filter bank consisted of 25 channels with centre frequencies equally spaced between 5 and 96 kHz on a logarithmic frequency axis^46^. The spectral transmission characteristics (Q10dB) of the filter bank were derived from a fit from distortion-product otoacoustic emissions (DPOAE) measurements^25^ (Q10dB values of 3.62 4.03 4.29 5.15 6.12 7.82 10.73 for frequencies between 10 and 70 kHz measured in 10 kHz steps). (3) A simplified version of non-linear transformations by the organ of Corti was implemented as half-wave rectification and exponential compression. The exponent was set to 0.4, following measures of cochlear compression^65^. (4) The temporal integration that arises from the generation of the inner hair cells’ receptor potential was implemented by a 1 kHz low-pass filter^46,66^, with a slope of 6 dB per octave. (5) In order to limit overall encoding accuracy of the model, auditory spectrograms (Figure S4) were energy-normalised and random noise with a fixed standard deviation of 3.1 (‘internal noise’) was added. (6) Finally, a decision device served as an optimal detector^67,68^. A formalized description of the decision device is given in the Supplementary Materials. The decision device operated under the following assumptions: First, the decision rule used by the bats is the maximum posterior decision rule, as this will result in the bat minimizing the probability of making a mistake. Second, based on the many presentations during training in the psychophysical 2-AFC paradigm, bats form a template of the auditory spectrogram that is associated with the rewarded stimulus (the original, non-phase-randomized version). Third, during each trial, the bat then compares the auditory spectrograms of both the original and the phase-randomized version with this template in both stimulus presentation intervals (Figure S5). During the simulation, we generated the template by averaging auditory spectrograms from 20 presentations of the rewarded stimulus (note that these 20 auditory spectrograms were not identical due to the added internal noise). The decision device chooses the auditory spectrogram that resembles the template the most (i.e., smallest Euclidean distance to template), as this maximises the probability of receiving a reward. Hence, we chose the interval resulting in the smallest Euclidean distance between the template and the auditory spectrograms generated from both intervals in each trial (n = 50). With this measure of discriminability^46^ we simulated the model’s discrimination performance as a function of phase-randomization window length.

The calibration of the model followed a previous study^69^. The model’s internal noise, i.e., the standard deviation of the added internal noise, was adjusted until the model performed at the same level as the bats in discriminating between two calibration signals with a 3 dB level difference^70^. As calibration signal, we used band-pass (5–80 kHz) filtered white noise of 130 ms length to match the mean duration and spectral range of the *P. discolor* communication calls used in the study. This detection threshold was used to determine the standard deviation of the added internal noise. Before being fed into the model, waveforms of the original call stimuli were normalised based on their root mean square amplitude (RMS). Phase-randomized versions were then scaled relative to their respective original stimulus.

#### Analysis of information contained in the P. discolor communication calls

A crucial question of our study is whether the threshold for discrimination of different phase-randomization window lengths (i.e., the amount of spectro-temporal integrity) can be related to information contained in the calls. In our case, the term “information” strictly refers to Shannon’s Mutual Information $MI$^22^. It is not synonymous with any semantical information that may be inherent to the communication calls.

In the current study, the maximal amount of $MI$ that can be communicated is 1 bit, because in a 2-AFC paradigm bats only have two equally probable options: the rewarded stimulus is presented either from the left or from the right loudspeaker. We can calculate $MI$ using the probability $P_{R}$ that the model’s decision device chooses the correct loudspeaker/stimulus (see derivation in the Supplementary Materials) given the calls emitted by both loudspeakers:

1 $$ \begin{aligned}   MI &  = H\left( p \right) - H\left( {p\left| S \right.} \right) \\     &  = 1 - \left( { - P_{R} *log_{2} \left( {P_{R} } \right) - \left( {1 - P_{R} } \right)*log_{2} \left( {1 - P_{R} } \right)} \right) \\  \end{aligned} $$

with $\user2{H}\left( \user2{p} \right)$ denoting the entropy of the distribution describing the probability that a given loudspeaker emitted the original call and $\user2{H}\left( {\user2{p}\left| \user2{S} \right.} \right)$ denoting the entropy of the distribution describing the probability that a given loudspeaker emitted the original call given the calls *S* emitted by both loudspeakers. The value of $\user2{P}_{{\user2{R~}}}$ can range between 0.5 and 1.0. Assuming a constant amount of internal noise influencing auditory periphery processing, $\user2{P}_{{\user2{R~}}}$ is influenced only by the spectro-temporal properties of the communication call fed into the model and by the window-length that is applied for the call’s phase-randomization.

### Psychophysics

#### Experimental setup

The experiments were performed on an open Y-maze inside a dark, echo-attenuated chamber (for details^71^). The loudspeakers (Peerless XT25SC40-04, Tymphany HK Limited, San Rafael, CA, USA) and food dispensers (custom-made) were mounted at the end of each arm of the maze. The experiment was observed from outside the chamber via an infrared camera (TV6819, Abus, Wetter, Germany). Stimulus presentation and data recording were controlled via a custom Matlab R2015 application and Soundmexpro plugin (192 kHz sampling rate; HörTech gGmbH, Oldenburg, Germany).

#### Psychophysical procedure

Two male bats were trained to discriminate an unaltered (original) stimulus from a phase-randomized (59.9 or 100 ms window length depending on individual stimulus) version of that same stimulus. Training/recording sessions (one to three per day) each lasted ten minutes. For fully trained bats, each potential recording session started with five to ten warm-up trials (using the easiest condition) to assess motivation. Bats needed to respond correctly to 4-out-of-5 or 8-out-of-10 trials, otherwise the session was not recorded. During a recording session, single trials were aborted when bats did not decide within 30 s after stimulus presentation onset*.* Bats were trained on five days per week, followed by a two-day break. The experiment followed a two-alternative, forced-choice paradigm (2AFC) with food reinforcement. Bats were presented subsequently with both the original and a phase-randomized version of the same original, with both the position of the original stimulus (left or right arm of the Y-maze) and the order of presentation (original stimulus first or second) pseudo-randomized^72^ from trial to trial. The onset of the second stimulus was 0.5 s after onset of the first and the presentation was repeated every two seconds for 30 s. The ten stimuli were played at RMS amplitudes of 60 ± 1 dB SPL (@ 10 cm; mean ± SD).

Stimulus presentation commenced (original and phase-randomized) once a bat perched in the starting area of the Y-maze. Bats had to identify and move towards the original stimulus and were consequently rewarded as soon as they reached the corresponding feeder. Once a bat’s performance reached > 70% correct choices on five consecutive days with the longest phase-randomization window (21.6, 59.9 or 100 ms depending on individual stimulus), the window length was decreased. Decreasing the window-length makes original and phase-randomized stimulus more similar to each other, increasing the difficulty of the detection task. Starting each data acquisition session with three consecutive trials presenting the longest window (see above), data acquisition proceeded by decreasing the window length until the bats could not detect the original stimulus at all, and then restarting again at very long window lengths until the daily sessions were completed. All five original stimuli were presented (together with their phase-randomized counterpart) the same number of times. However, since single trials could be aborted, the actual proportion of each of the five stimuli in the recorded trials varies slightly. Testing was completed when at least 50 trials (~ ten trials per stimulus) were obtained per window length and bat.

#### Psychophysical data analysis

Percentage correct performance of the animals as a function of window length was determined by cubic smoothing spline interpolation (*csaps* function, Matlab 2020a). The value of this fit at 66% correct performance was taken as the overall psychometric threshold (*fnval* function, Matlab 2020a; 66% for *p* < 0.01 in a binomial test, 50 trials), which still enables a bat to reliably discriminate the original stimulus from the phase-randomized stimulus.

### Neurophysiology

#### Surgery

The surgical procedures are described in detail in a previous study^24^. One male and three female bats were anaesthetized with a combination of medetomidine (Dorbene, Zoetis, Parsippany, USA), midazolam (Dormicum, Hoffmann-La Roche, Basel, Switzerland) and fentanyl (Fentadon, Albrecht GmbH, Aulendorf, Germany) at a dosage of 0.4, 4.0 and 0.04 µg/g body weight, respectively. Anaesthesia was maintained through additional injections containing two-thirds of the initial dose every 1.5 h. To prevent drying, the bat’s eyes were covered with a vitamin-A cream (VitA POS, Ursapharm, Saarbrucken, Germany) during anaesthesia. The scalp was opened along the midline, the skull surface freed from tissue, and a small metal tube and microglass composite were used to fix the skull to the stereotaxic device. Details on reconstructing the recording sites are described elsewhere^73^. In brief, the characteristic profile lines of the skull were scanned in the parasagittal and frontal planes and digitally fitted to a standardized skull profile in a standardized coordinate system.

Anaesthesia was antagonized with a mixture of atipamezole (Alzane, Novartis, Basel, Switzerland), flumazenil (Flumazenil HEXAL, Hexal AG, Holzkirchen, Germany) and naloxone (Naloxon-ratiopharm, Ratiopharm GmbH, Ulm, Germany) injected subcutaneously (2.5, 0.5 and 1.2 µg/g body weight, respectively). An analgesic (0.2 µg/g body weight; Meloxicam, Metacam, Boehringer-Ingelheim, Ingelheim am Rhein, Germany). Antibiotics (0.5 µg/g body weight; enrofloxacin, Baytril, Bayer AG, Leverkusen, Germany) were administered for four postoperative days to alleviate postoperative pain and prevent infection, respectively.

#### Acoustic stimulation

All acoustic stimuli were computer-generated (Matlab), digital–analog converted (RX6, Tucker Davis Technologies (TDT), Gainesville, USA; sampling rate 195,312 Hz), attenuated (PA5, TDT), amplified (AX-396, Yamaha Music Foundation, Tokyo, Japan) and presented via a free-field loudspeaker (R2904/700,000, Scan-Speak, Videbæk, Denmark) which had been calibrated for linear frequency response between 1 and 95 kHz. The loudspeaker was positioned contralaterally to the recording site, at ~ 30° off the midline at ~ 20 cm distance.

#### Electrophysiological recordings

The experiments commenced two days after initial surgery. Recording sessions took place three days a week for up to eight weeks (with at least one day off between consecutive experiments) and could last up to five hours per day. After initial surgery, experiments were conducted in a sound-attenuated heated (~ 35 °C) chamber. Extracellular recordings were made with parylene-coated tungsten microelectrodes (5 MΩ impedance, Alpha Omega GmbH, Ubstadt-Weiher, Germany) in anesthetized bats. Note that responses recorded from cortical units under the applied anaesthesia regime reflect the behavioural performance of *P. discolor* well^74^. Dorso-ventral (DV) electrode penetrations in the auditory cortex (AC) were run obliquely to the brain surface with different medio-lateral (ML) and rostro-caudal (RC) angles. The electrode signal was recorded using an analog–digital converter (RA16, RX5; TDT), sampling rate 25 kHz, band-pass filter 400–3000 Hz) and BrainWare (TDT). The action potentials were threshold-discriminated and saved for offline analysis. Spike discrimination was done either by appropriate thresholding during recording or by off-line 2D-clustering of action-potential waveforms by negative/positive peak amplitude (Brainware analysis tools). As it was not always possible to isolate the activity of a single neuron, the term ‘unit’ will be used in the following to describe the activity of one neuron to clusters of three neurons recorded at a distinct recording site. In *P. discolor*, such mixtures of single-neuron and multi-unit cluster recordings from the auditory cortex are suited to predict behavioural performance in acoustic discrimination tasks (e.g.^45^).

To search for acoustically-driven neural activity, either a natural echolocation call (downward frequency-modulated, multiharmonic, main energy between 40 and 90 kHz, duration ~ 1.2 ms) or an aggression call (frequency- and amplitude-modulated, main energy between 0 and 20 kHz, duration ~ 170 ms) was presented periodically at a repetition rate of 2 Hz. During the search, the sound pressure levels (SPL) of the stimuli were varied while the neural activity was monitored visually and acoustically by the experimenter.

Once an adequate unit was found, we first measured its basic response properties. We established a frequency–response curve by presenting pure-tone stimuli and recording the neuronal response in a 250 ms response window beginning with stimulus onset. The stimuli were preceded by 50 ms silence. Each pure tone was 20 ms long, frequencies ranged from 5 to 80 kHz (logarithmically spaced in 1/8 octave steps) and SPL ranged from 15 to 80 dB re 20 µPa. Each stimulus was presented in random order and repeated ten times at a repetition rate of 2 Hz.

Subsequently, the five unaltered (original) stimuli were presented in random order with 20 repetitions each (rep. rate ~ 0.7 Hz) at ~ 15–20 dB above characteristic frequency (CF) threshold. The preferred original stimulus, which caused the strongest neuronal response, was identified and thereafter employed for acoustic stimulation together with its phase-randomized counterparts (see section “Stimuli”), using the same stimulation parameters as before.

#### Verification of recording sites

After completion of the experiments, a neuronal marker (BDA 3000, Sigma-Aldrich, St-Louis, USA; 5% in phosphate buffer) was pressure-injected (Nanoliter 2010 injector, World Precision Instruments, Sarasota, FL, USA) into the brains in order to reconstruct the position of the recording sites in standardized stereotactic coordinates^73^ of a brain atlas of *P. discolor*^75,76^. Subsequently, the bats were euthanized with an intraperitoneally applied lethal dose of pentobarbital (Narcoren, Boehringer-Ingelheim, 0.16 mg/g bodyweight) and transcardially perfused with 4% paraformaldehyde.

#### Data analysis

The unit’s spike responses to the stimulus set, i.e. the original stimulus with strongest neuronal response and the corresponding phase-randomized versions, were displayed as peri-stimulus time histograms (PSTH, bin width 1 ms) and raster plots. Few units showed spontaneous activity and, when present, the spontaneous spike rate was very low (< 10 spikes/s). The mean spike rates of the neuronal responses were calculated over an individually set response window, which began with the onset of stimulus presentation, and ended when the unit’s neuronal discharge pattern reached spontaneous level.

We formed predictions about spectro-temporal resolution thresholds by generating neurometric functions. A neurometric function reflects the probability that an ideal observer could accurately discriminate phase-randomized versions of a signal from the original signal based on responses like those recorded from the units under study. A receiver operating characteristics (ROC) analysis^67,74,77^ was performed by generating ROC curves for the comparison of each signal condition (increasingly phase-randomized versions of a call) and the standard condition (non-phase-randomized version of a call). The ROC curve shows the probability that both the response in a signal condition and the response in the standard condition exceed a certain threshold (spikes per stimulus in increments of one spike). This probability was plotted as a function of the height of the threshold. From there, the (neural) percentage of correct discrimination for each signal condition (i.e., the neurometric function) was generated by calculating the area under the ROC curve. The so-obtained neurometric function was fitted with a cubic smoothing spline interpolation (*csaps* function, Matlab 2020a), and the 75% correct threshold (*fnval* function, Matlab 2020a; binomial test, 20 trials, *p* < 0.01) was calculated when applicable. After analysing the ROC curves for each unit, we calculated the ROC curve for the population of units with significantly decreasing spike count as a function of randomization-window-length. This procedure was preferred over calculating a mean response, as the ROC curve should reflect the decision of an ideal observer looking at this neuronal population as a whole.

## Supplementary Information

Supplementary Information.

## Data Availability

The datasets generated and/or analysed during the current study are available from the corresponding author on reasonable request.

## Abbreviations

2AFC: 2-Alternatives, forced-choice
AC: Auditory cortex
ADF: Anterior dorsal field
AM: Amplitude modulation
BF: Best frequency
CF: Characteristic frequency
DV: Dorso-ventral
ML: Medio-lateral
PDF: Posterior dorsal field
PSTH: Peri-stimulus time histogram
RC: Rostro-caudal
RMS: Root mean square
ROC: Receiver operating characteristics
SAM: Sinusoidal amplitude modulation/sinusoidally amplitude modulated
SFM: Sinusoidally frequency modulated
SPL: Sound pressure level
