# Supplementary material for: Communication breakdown: Limits of spectro-temporal resolution for the perception of bat communication calls
Source: Sci Rep. 2021 Jul 1;11:13708. doi: 10.1038/s41598-021-92842-4 (PMC8249457; doi:10.1038/s41598-021-92842-4)
Supplement: Supplementary file 1 — Supplementary Information. [file 41598_2021_92842_MOESM1_ESM.docx]

**Communication Breakdown or Limits of spectro-temporal resolution for perception of bat communication calls**

*S. G. Hörpel, A. L. Baier, H. Peremans, J. Reijniers, L. Wiegrebe, U. Firzlaff*

Supplementary Information

Figures S1, S2, S3, S4, S5

Formal description of model decision device

***Figure S1: Original and phase-randomised appeasement calls***

***A****) The spectrograms of the five original appeasement calls used in this study illustrate strong frequency modulations (FFT size 1024 samples, overlap 1020 samples).* ***B****) The spectrograms of one of the original calls (highlighted in red) and its seven phase-randomized versions (highlighted in magenta) exemplify the effect of increasing randomization-window lengths ranging from 1 ms to 22 ms (top to bottom, cf. panel D).* ***C****) The waveforms of the signals in (B) illustrate the decrease in envelope entropy with increasing randomization-window length (top to bottom) All calls were normalized to RMS amplitude.* ***D****) The magnitude spectra of the signals in (B) reveal no strong effect of the phase-randomization. Numbers depict randomization-window lengths ranging from 1 ms to 22 ms. The figure was created using Matlab (2020a; MathWorks, Natick, USA).*

**
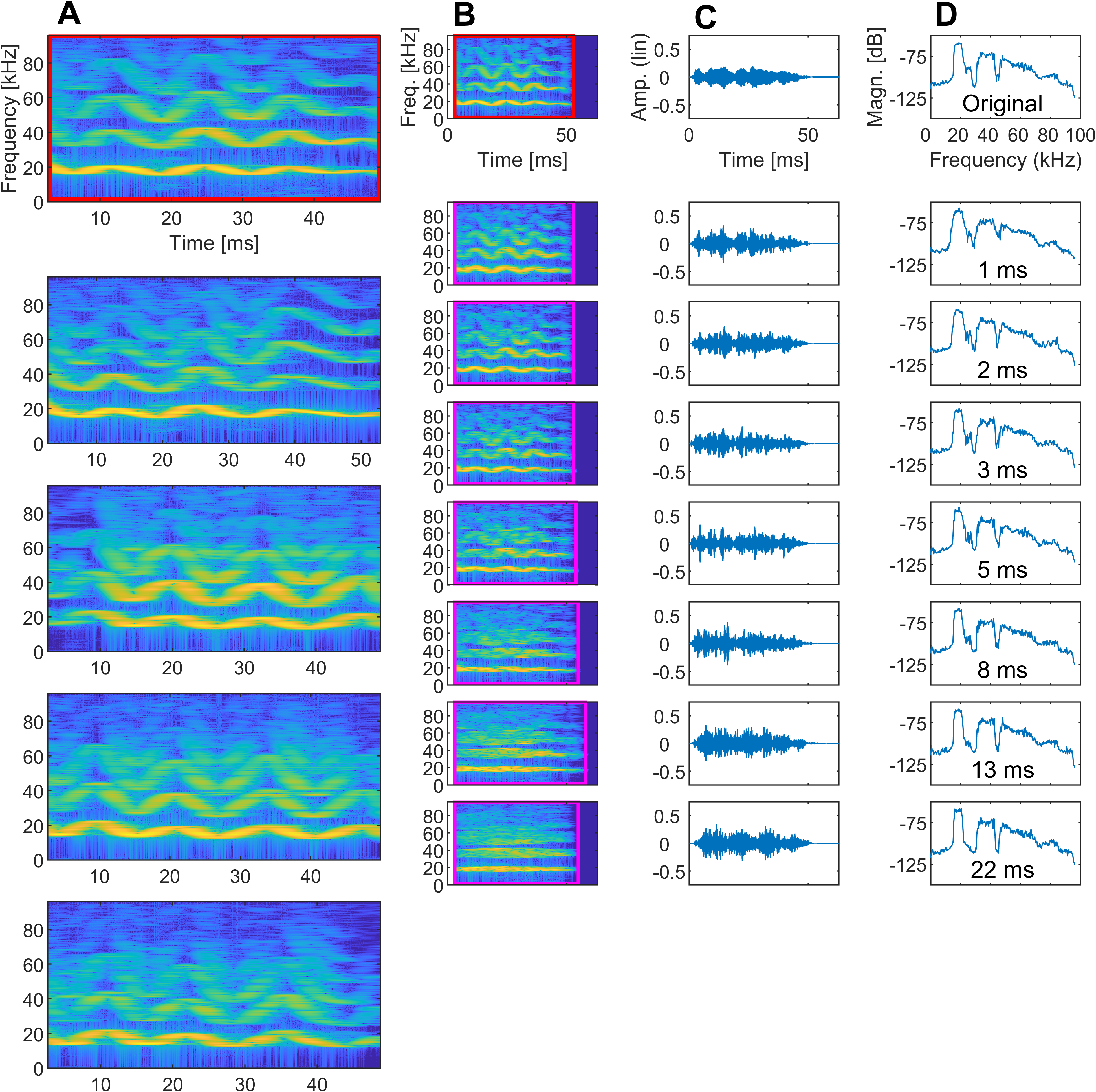
**


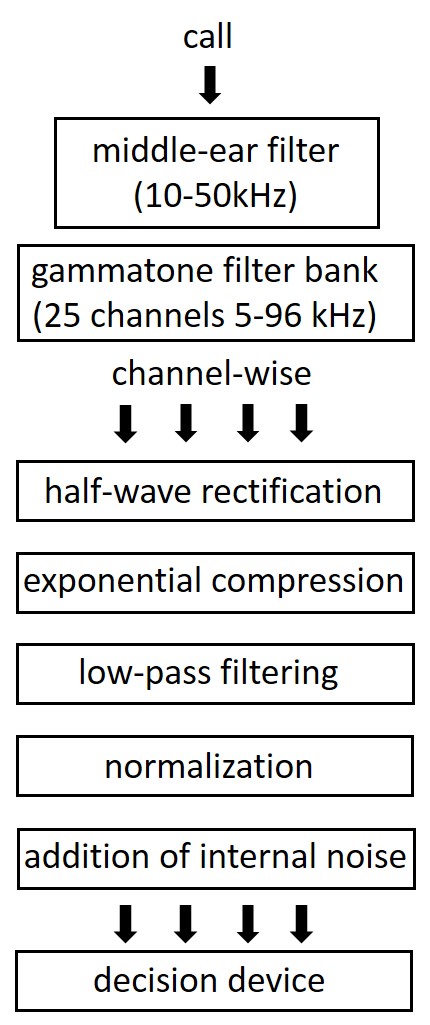


***Figure S2: Flowchart representation of the model***

*The input to the model is a communication call of P. discolor. After band-pass filtering filtering (1st-order Butterworth) to emulate the transfer characteristics of the bat middle ear, frequency-to-place conversion of the inner ear is achieved with a series of 4th-order gamma-tone filters (25 channels, centre frequencies logarithmically spaced from 5-96 kHz). The non-linear transformations by the organ of Corti are realized as half-wave rectification and exponential compression and temporal integration arising from the generation of the inner hair cells’ receptor potential is emulated by a 1 kHz low-pass filter with a slope of 6 dB/octave (1st-order Butterworth). Finally, the resulting auditory spectrograms are energy-normalized and random noise is added to limit overall model accuracy. A decision device serves as optimal detector to determine* discrimination performance *of the model.*


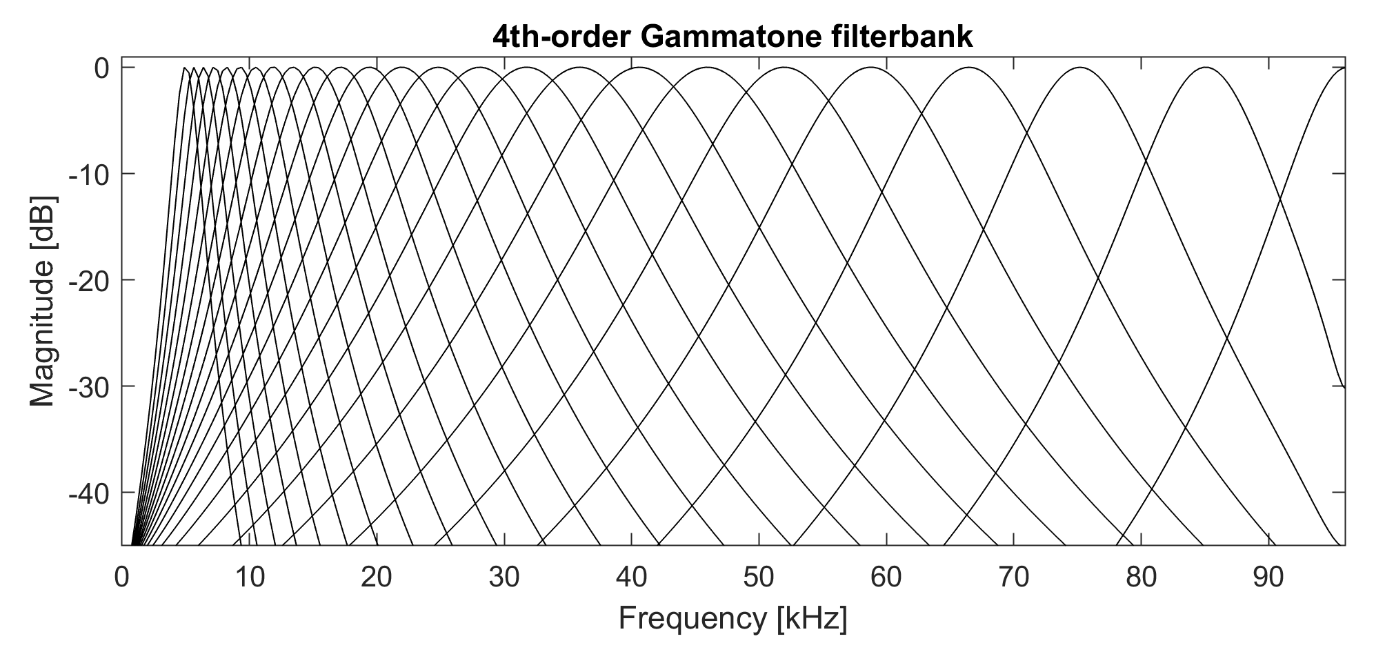


***Figure S3: Visualization of the gamma-tone filter bank***

*The frequency-to-place conversion of the inner ear was emulated with a series of 4th-order gamma-tone filters. The filter bank consisted of 25 channels with centre frequencies equally spaced between 5 and 96 kHz on a logarithmic frequency axis. The spectral transmission characteristics (Q10dB) of the filter bank were derived from a fit from distortion-product otoacoustic emissions (DPOAE) measurements (Wittekind et al. 2005; Q10dB values of 3.62 4.03 4.29 5.15 6.12 7.82 10.73 for frequencies between 10 and 70 kHz measured in 10 kHz steps).*


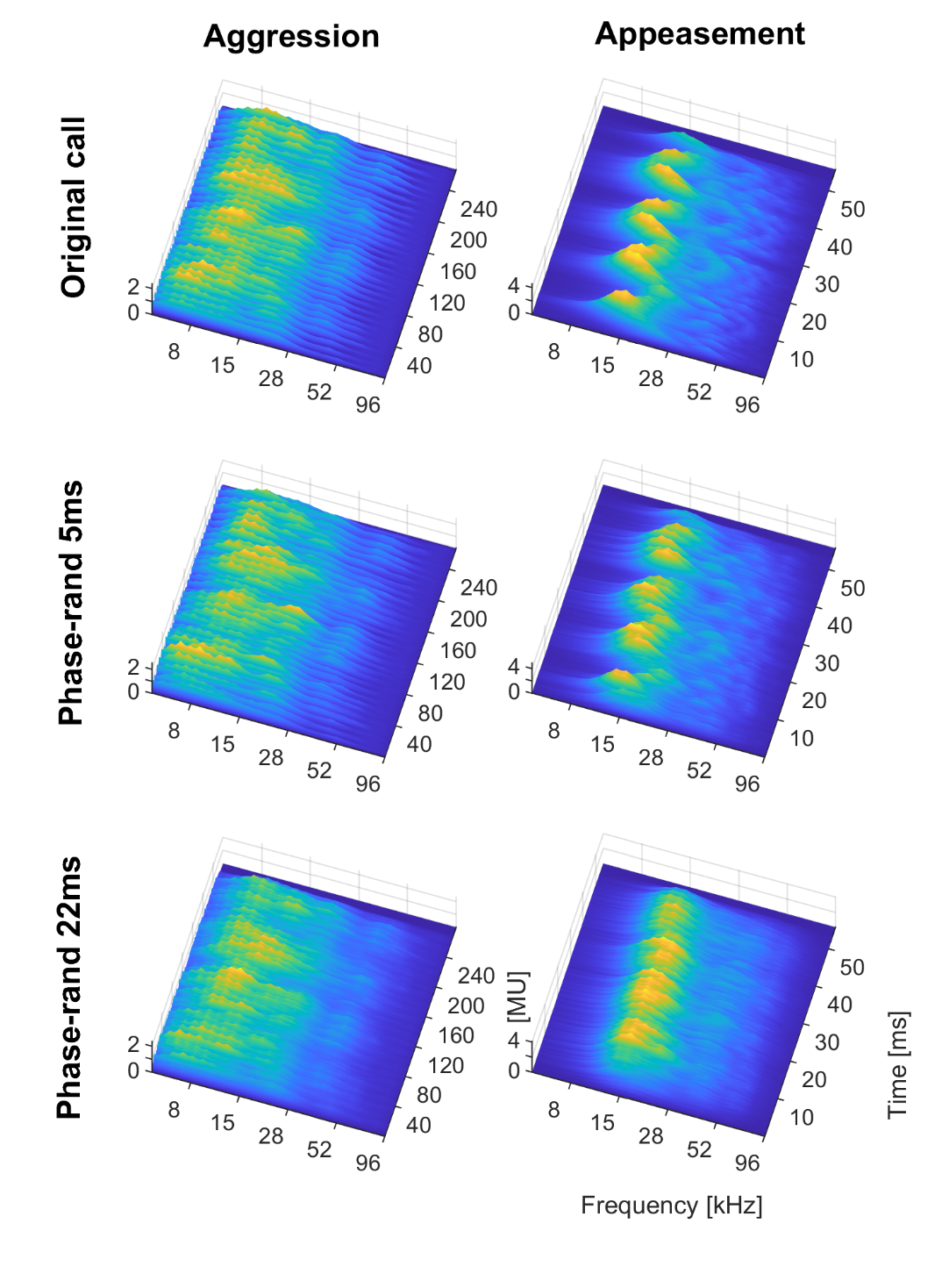


***Figure S4: Auditory spectrograms of aggression and appeasement calls***

*Exemplary auditory spectrograms of an aggression and an appeasement call illustrating the distorting effect of phase-randomization for two different window-lengths. Spectro-temporal modulation patterns of calls are increasingly blurred in the different frequency channels. MU= model units. The figure was created using Matlab (2020a; MathWorks, Natick, USA).*

**
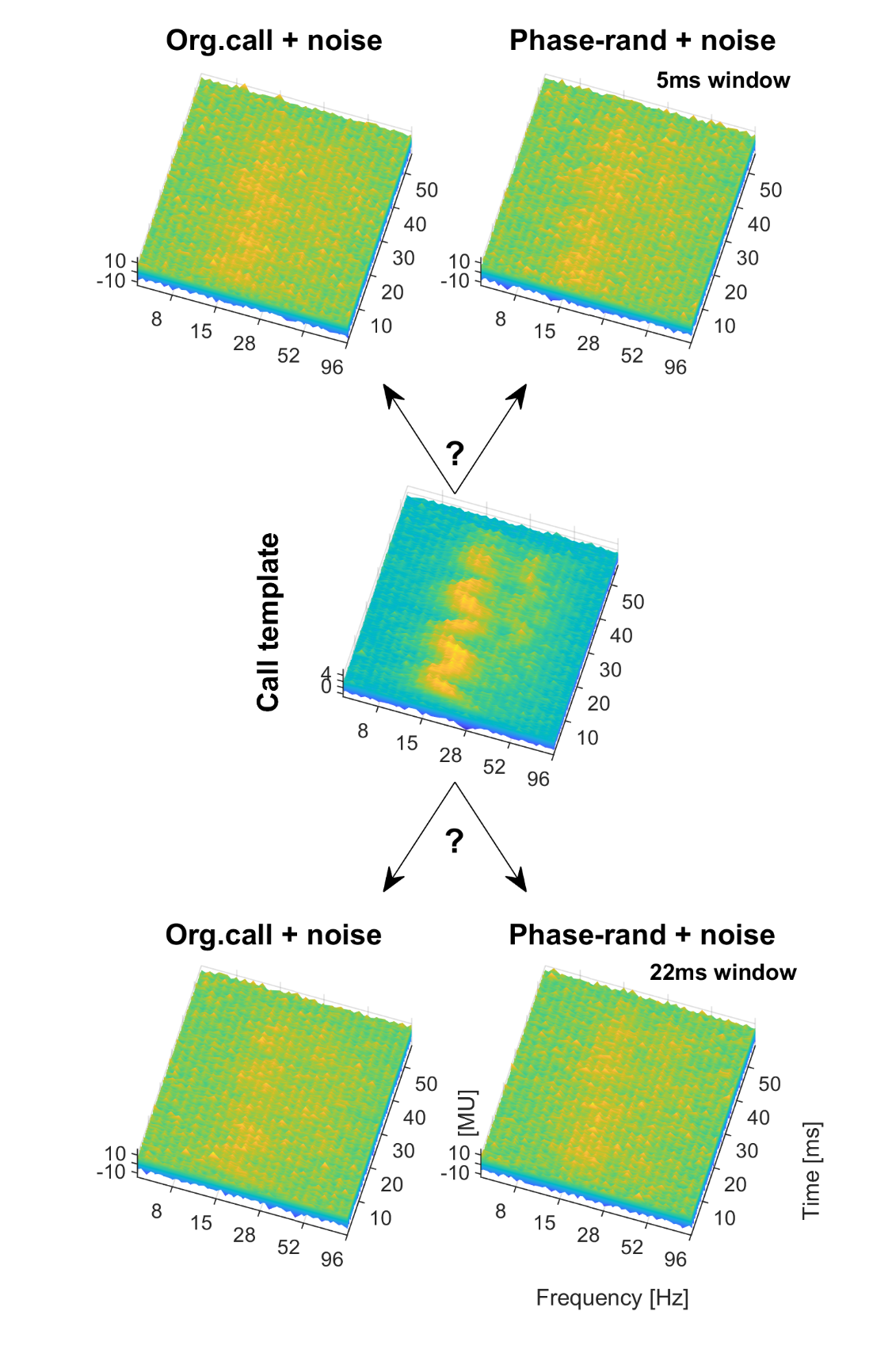
Formal description of the model decision device**

***Figure S5: Exemplary auditory spectrograms with added internal noise***

*Random noise (“internal noise”) was added to the auditory spectrograms (AS) to limit overall model accuracy before AS were passed to the decision device. The decision device was required to assign either the original or manipulated calls to the call template. Examples for two different phase-randomization window lengths are shown. Discrimination is more difficult for longer window-lengths as the spectro-temporal structure is more blurred. Note that noise was always freshly generated for each trial. Therefore, AS for the original call plus noise are not exactly the same for the two examples in the figure. For further details see Methods-Section. The figure was created using Matlab (2020a; MathWorks, Natick, USA).*

**Formal description of the decision device:**

**The bat has to decide whether the right or the left loudspeaker is the one emitting the original call with both loudspeakers having equal probability** $P\left( R \right)=P\left( L \right)=0.5$ **of emitting the original call during the experiments. We will assume in this derivation that the right loudspeaker is the correct decision, i.e. the signal received from the right loudspeaker is the original call** $s_{R}=Call$ **and the signal received from the left loudspeaker is a distorted call** $s_{L}=Distorted Call$**. Using Bayes formula, the probability** $P_{R}$ **that the right loudspeaker is emitting the original call given the received signals from both loudspeakers can then be written as**

$$P_{R}=P\left( R\left| \left[ s_{R},s_{L} \right] \right. \right)=\frac{p(\left[ s_{R},s_{L} \right]\left| R \right.)P(R)}{p\left( \left[ s_{R},s_{L} \right]\left| R \right. \right)P\left( R \right)+p(\left[ s_{R},s_{L} \right]\left| L \right.)P(L)}$$

**with** $P(\left[ s_{R},s_{L} \right]\left| X) \right.$ **denoting the likelihood of receiving signals** $\left[ s_{R},s_{L} \right]$ **when loudspeaker *X* is emitting the original call. A similar expression can be derived for the probability** $P_{L}$ **that the left loudspeaker is emitting the original call given the received signals from both loudspeakers, but as there are only two,** $P_{L}=1-P_{R}$ **. As the emissions of the loudspeakers are independent, we write** $p(\left[ s_{R},s_{L} \right]\left| R \right.)=p(\left[ s_{R} \right]\left| R \right.)p(\left[ s_{L} \right]\left| R \right.)$**. In accordance with the model presented in the main text, we choose** $p(\left[ s_{R} \right]\left| R \right.)$ **as Gaussian noise superimposed on an internal template representing the original call** $s_{M}=Memorised Call$ ^1^ **learned by the bat during training, or**

$$p(\left[ s_{R} \right]\left| R \right.)=\frac{e^{-\frac{1}{2}\left( s_{R}-s_{M} \right)^{T}\Sigma^{-1}\left( s_{R}-s_{M} \right)}}{\sqrt{\left( 2\pi\right)^{N}\left| \Sigma\right|}}$$

**Assuming the time-frequency samples of the auditory spectrogram to be independent the covariance matrix can be written as** $\Sigma=\sigma^{2}\cdot I,$ **with *I* the identity matrix and** $\sigma^{2}$ **the variance of the internal noise as determined by the calibration procedure referred to in the main text. Hence, we get**

$$p(\left[ s_{R} \right]\left| R \right.)=\frac{e^{-\frac{1}{2\sigma^{2}}\sum_{t,f} \left( s_{R}\left( t,f \right)-s_{M}\left( t,f \right) \right)^{2}}}{\sqrt{\left( 2\pi\right)^{N}}\sigma^{N}}$$

**where the summation ranges over the** $N$**time-frequency samples of the auditory spectrogram. Note that this makes** $p\left( \left[ s_{R} \right]\left| R \right. \right)$ **a monotonically decreasing function of the Euclidean distance between the template** $s_{M}$ **and the signal received from the right loudspeaker** $s_{R}$**. In the same way, we can derive the expression for the likelihood of receiving the signal** $s_{L}$ **from the left speaker** $p\left( \left[ s_{L} \right]\left| L \right. \right)$ **when the left loudspeaker is emitting the original call**

$$p(\left[ s_{L} \right]\left| L \right.)=\frac{e^{-\frac{1}{2\sigma^{2}}\sum_{t,f} \left( s_{L}\left( t,f \right)-s_{M}\left( t,f \right) \right)^{2}}}{\sqrt{\left( 2\pi\right)^{N}}\sigma^{N}}$$

**The term** $p(\left[ s_{L} \right]\left| R \right.)$ **on the other hand denotes the likelihood of receiving a particular distorted version of the call from the left loudspeaker** $s_{L}$ **when the right loudspeaker is emitting the call. However, to use this information the bat would need to know what types of distortions are more likely than others. It is not clear whether the bat can learn this from the training phase. Hence, we will assume that the bat treats all distortions as equally likely. Following an analogous reasoning for** $p(\left[ s_{R},s_{L} \right]\left| L \right.)$ **we can rewrite the equation above as**

$$P_{R}=\frac{p(\left[ s_{R} \right]\left| R \right.)}{p\left( \left[ s_{R} \right]\left| R \right. \right)+p(\left[ s_{L} \right]\left| L \right.)\cdot\frac{p(\left[ s_{R} \right]\left| L \right.)}{p\left( \left[ s_{L} \right]\left| R \right. \right)}}$$

**Assuming the bat maximizes the posterior probability of its choice, thereby minimising unrewarded errors, the bat will choose the right loudspeaker whenever** $P_{R}>P_{L}$

$$\frac{P_{R}}{1-P_{R}}=\frac{p(\left[ s_{R} \right]\left| R \right.)}{p(\left[ s_{L} \right]\left| L \right.)\cdot\frac{p(\left[ s_{R} \right]\left| L \right.)}{p\left( \left[ s_{L} \right]\left| R \right. \right)}}>1$$

**or equivalently**

$$\frac{p(\left[ s_{R} \right]\left| R \right.)}{p(\left[ s_{L} \right]\left| L \right.)}>\frac{p(\left[ s_{R} \right]\left| L \right.)}{p\left( \left[ s_{L} \right]\left| R \right. \right)}$$

**If we assume that the bats treat all distorted calls as equally likely,** $p\left( s_{R}\left| L \right. \right)=p\left( s_{L}\left| R \right. \right)$**, this decision rule reduces to the classic maximum likelihood rule as used in the main text, i.e. choose the right loudspeaker whenever**

$$\frac{p(\left[ s_{R} \right]\left| R \right.)}{p(\left[ s_{L} \right]\left| L \right.)}>1$$

**However, it should be noted that a bias can be introduced with respect to this rule by the bat learning about the likelihood of the distortions.**

**Replacing both likelihoods by the expressions derived above it can be seen that the proposed decision rule effectively compares the Euclidean distances between the memorised call** $s_{M}$ **and the signals received from the left and right loudspeaker, respectively** $s_{L}$ **and** $s_{R}$**, and chooses the loudspeaker emitting the signal at the smallest Euclidean distance from the memorised call.**

**References**

1. Dau, T., Püschel, D. & Kohlrausch, A. A quantitative model of the ‘“effective”’ signal processing in the auditory system. I. Model structure. *J. Acoust. Soc. Am.* **99**, 3615–3622 (1996).
